# The human pangenome reference anticipates equitable and fundamental genomic insights

**DOI:** 10.1016/j.xgen.2023.100360

**Published:** 2023-07-12

**Authors:** Kelly A. Frazer, Nicholas J. Schork

**Affiliations:** 1 Department of Pediatrics, University of California San Diego, La Jolla, CA 92093, USA; 2 Institute of Genomic Medicine, University of California San Diego, 9500 Gilman Dr., La Jolla, CA 92093, USA; 3 Quantitative Medicine and Systems Biology Division, Translational Genomics Research Institute, Phoenix, AZ 85004, USA; 4 Departments Molecular and Cellular Biology and Population Sciences, City of Hope National Medical Center, Duarte, CA 91010, USA

## Abstract

For the past few years, researchers in the Human Pangenome Reference Consortium (HPRC) have been working to catalog almost all human genomic diversity. Frazer and Schork preview an article recently published in *Nature*, “A draft human pangenome reference,”^1^ which represents the initial release of 47 fully phased diploid assemblies of genomes of individuals with diverse ancestries.

## Main text

The sequencing of the human genome more than 20 years ago was met with great enthusiasm. The belief that an understanding of the overall organization of the human genome would lead to insights into the origins of humans, genetically mediated phenotypic diversity, and new and better medicines motivated many additional initiatives. These additional initiatives, which include the International Hapmap Project,^2^ the 1000 Genomes Project,^3^ The Cancer Genome Atlas (TCGA) Project,^4^ genome-wide association studies (GWASs) of all sorts,^5^ and related DNA sequencing projects, have a focus on exploring and characterizing inter-individual genomic variation—with that in mind, if we can understand the genetic differences between individuals, with and without a disease or that do and do not respond to a drug, we can identify not only individuals at risk for disease more reliably but also better ways of treating people with diseases.

At least three issues arose, however, as these projects unfolded— all of which were anticipated to some degree. First, the continued reliance and use of “convenience” samples (i.e., available samples from biorepositories and legacy cohorts) resulted in more genomic information on individuals of largely European ancestry, leading to overt blind spots in the characterization of global human genetic variation, especially variation that may be clinically meaningful. Second, although the costs of DNA sequencing have gone down exponentially in the last two decades, what we have learned about the complexity of the human genome is such that characterizing all the phenomena needed to fully understand individual human genomic variation (e.g., structural variations, repeated sequences, individual chromosomal segment pairs in diploid contexts, etc.) is still technically challenging. These technical issues have been overcome to some degree with the efforts described in the recent publication by the Human Pangenome Reference Committee (HPRC).^1^ Third, the use of DNA sequencing in a wide variety of research contexts has revealed both complex historical and recent migrations and mixing of populations, resulting in many admixed individuals with genomes that harbor sequence inherited from more than one ancestral population and complex molecular biology and pathology—for example, the role haplotype-specific gene expression and DNA modifications play in mediating pathogenic processes in specific cellular contexts that are difficult to model in genetic studies. We comment on each of these three issues below, emphasizing the potential solutions provided by the construction of the pangenome and where the genomics community can pick up going forward.

**Figure 1:**
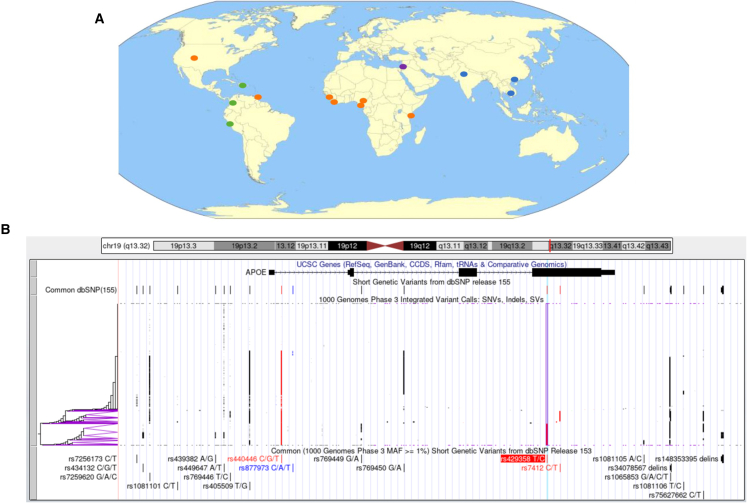
A pangenome composed of 47 phased genomes (A) Selection of the 47 HPRC samples. There were 7 geographical locations that individuals with African ancestry (orange circles) were collected, 3 for individuals of Americas ancestry (green circles), 3 for Asia ancestry (blue circles), and one of Europe ancestry (purple). (B) UCSC Genome Browser (https://genome.ucsc.edu) view of haplotypes possessed by individuals of different ancestries in the APOE gene region based on the 1000 Genomes data. The top and bottom panels depict the overall structure of the region with, e.g., gene boundaries and SNP positions for the APOE gene. The middle panel provides haplotypes clustered by similarity across individuals with diverse ancestries represented in the 1000 Genomes Project, where vertical lines represent alternate nucleotides from the GRch37/hg19 reference genome, red represents non-synonymous variants, blue represents UTR/noncoding variants, and black represents other variants. The purple lines represent the variant (rs429358) used for sorting the haplotypes and that defines the well-studied APOE4 allele. The variation depicted reflects the evolutional and functional diversity of different haplotypes possessed by individuals associated with diverse populations of the type the HPRC is seeking to better characterize.

Over the past 20-plus years, the reliance on the use of convenience samples for sequencing efforts associated with many contemporary projects has led to disparities in the amount of genomic information available on individuals of different ancestries. This has had the consequence of leading to disparities in health care enhanced by genomic information, since, e.g., for non-Europeans, clinically actionable mutations may not be recognized^6^ and genetic risk scores assessing susceptibility may not be available or validated.^7^ These health inequities are technical in nature and different than social problems creating disparities in care (e.g., socioeconomic status, education, environmental exposures, employment, etc.). While the root causes of health inequities are broad, release of the pangenome has shown that a concerted effort in the scientific community could, in a matter of a few years, improve our understanding of diverse genomes, which would eliminate the technical barriers impeding the delivery of genetic diagnostics and key clinically actionable insights applicable to all. In this light, the 47 individuals in the recent HPRC release were selected from 14 geographical locations to represent individuals with African, American, Asian, and European ancestries (Figure 1A). By including genomes from individuals selected to represent people across the world, the HPRC’s efforts attempt to make genome research more inclusive and ultimately more equitable. Looking ahead, the plan to expand the pangenome from 47 genomes to 350 even more diverse genomes by 2024 indicates the commitment of researchers to further uncover the complexities of human genetic diversity. The selection of the next 303 HPRC samples is crucial for ensuring that it benefits the translation of genetics research into the clinic for individuals regardless of their ancestral backgrounds. There are, however, communities noticeably absent in the current draft pangenome (e.g., Native American) as justifiable yet complex ethical and social issues need to be worked out prior to the inclusion of these groups, although the payoff would be worth the effort.

The importance of good reference genomes cannot be underestimated in contemporary genomics research, as they serve as coordinate systems for genomic analyses. The shortcomings of the current reference genomes include that they are haploid and largely based on the linear DNA sequence from single individuals. These limitations combined with their primary use for mapping sequencing reads from technologies that generate short stretches of DNA have resulted in most genomic analyses focusing on a narrow class of genetic variation (e.g., single nucleotide variants). The effort to construct the pangenome, which involved assembling, *de novo*, genome sequences from 47 individuals, is an overt attempt to overcome these limitations. The construction of the pangenome exploited an approach that not only enabled its efficient and reliable construction but more accurately reflects underappreciated aspects of the human genome. Simply put, humans are diploid and genomic variation is complex, involving structural variations, complex repeated and inverted sequences. Each cell in the human body harbors two nuclear genomes, one inherited from each parent. These two genomes contribute to the homologous chromosomal pairs making up the 22 autosomal pairs and the sex chromosomes defining the diploid genome. The nucleotide content of each chromosomal homolog in the 22 autosomal pairs and the sex chromosomes is itself made up of unique stretches of DNA sequence that vary between individuals because of mutations and recombination events occurring in meiosis involving the homologous chromosomal pairs possessed by each parent at the time of the formation of gametes ultimately transmitted to an individual offspring. Thus, human genomes are basically an amalgamation of stretches of DNA sequences harboring sequential variants (haplotypes) whose nucleotide content can affect all sorts of *cis* (i.e., involving genomic elements on the same chromosomal homolog) and *trans* (i.e., involving genomic elements on different chromosomal homologs) interactions that impact gene function in a variety of ways.^8^^,^^9^ The HPRC team acknowledged this, recognizing that knowing precise DNA sequences, in their “phased” (haplotyped) form, is an integral part, but not the *only* part, of enabling complicated sets of queries that need to be pursued to truly understand human genomic biology. Consider Figure 1B, which depicts haplotype variation in the well-studied APOE gene region that encompasses coding and noncoding variants. The individual and collective *cis*- vs. *trans*-acting functional effects of these variants need further characterization. Thus, by recognizing and exploiting the diploid nature of the human genome, the HPRC efforts pave the way for more sophisticated, higher-resolution investigations in molecular, population, and evolutionary biology.^8^^,^^9^^,^^10^

Given the motivation for the HPRC, which was to acknowledge and accommodate human global genome diversity, its pangenome construction via the exploitation of phased genomes and its potential contributions to helping sort out fundamental aspects of human molecular biology, a good question is: what is next? Obviously, the expansion of the pangenome to generate sequence data from individuals with more diverse ancestries to overcome technical disparities in genomic medicine is crucial. However, the core of the HPRC—the 47 *de novo* assembled and phased genomes that did not require a reference genome for their construction—suggests that maybe the community should simply move away from the use of reference genomes and focus on the efficient, reliable, and routine *de novo* assembly of individual genomes. Moreover, the content of such genomes (e.g., haplotype pairs encompassing defined genic regions possessed by different individuals) could potentially be used to replace the current ancestral designations (African American, Hispanic, American Indian, European) of individuals who are commonly admixed. For example, the comparison of haplotypes possessed by individuals to examine their commonalities, in terms of nucleotide content and the phenomena that could have led to a lack of commonalities, as a guide for assessing genomic similarity and ancestry could complement or replace the comparison of individual genomes to often idealized, vague, and socially complex ancestral reference individuals.

Overall, while the full extent of the impact of the pangenome is yet to be determined, it is evident that the efforts surrounding its construction provide a significant step forward, enabling genomics research that can provide more fundamental insights into human molecular biology and do so in a more equitable way.
